# Eruption of Two Inferior Central Incisor Teeth Second Day after Birth, Their Extraction Followed by Hemorrhage and Death

**Published:** 1877-09

**Authors:** M. Th. David


### ERUPTION OF THE TWO INFERIOR CENTRAL INCISOR TEETH, SECOND DAY AFTER BIRTH; THEIR EXTRACTION FOLLOWED BY HEMORRHAGE AND DEATH.

BY M. TH. DAVID.

[*Translated from the Gazette des Hopiteaux, for the Dental Register.*]

The child at birth was possessed of an ordinarily good constitution. Two days after, it was noticed that the points of the two inferior central incisors were visible about the margin of the gums. These teeth continued to emerge during the following days, and gradually loosened until they seemed to be supported only by a kind of neck, which allowed free movement in every direction. As they seemed ready to fall out, and troubled the child in nursing, the family physician, who was one of the masters in the Paris Hospital, believing their removal necessary, extracted them on the 20th of January, that is, to say, three weeks after the birth of the child. The operation was soon followed by severe hemorrhage, which could only be stopped by digital compression which was continued during the whole night.

The next morning the bleeding which had for a short time subsided, recommenced, and continued the greater part of that day. Napkins were used to staunch the blood, which was in quantity really alarming. At two o'clock, one of our young and able practitioners was sent for, who immediately cauterized the interior of the alveoli, from whence the blood came with an olivary cautery about the size of a pea. This checked the hemorrhage for some hours, when it again commenced with the same intensity.

The next day (the 22nd) cauterization was again resorted to in the same manner, and the flow of blood was stopped. Five days afterwards, however, it recommenced, necessitating a third application of the cautery. This last, unfortunately, resulted in the complete destruction of the gum and alveolar margin in the part corresponding to the two incisors. During this operation, several hard and brilliant bodies were seen beneath the wound, which were none other than the crowns of several permanent teeth in process of formation.

Notwithstanding this last application of the cautery, the hemorrhage reappeared, and continued until the 10th of February, when we were again sent for, but the child was then dying.

Far from us the thought of criticising the practice adopted in this case by our honorable *confreres*. We will then neither discuss the expediency of extracting these loosened teeth, nor the means employed in fighting against a hemorrhage of such an obstinate character. We are of the opinion that in this unfortunate affair, the extraction of the teeth only anticipated their spontaneous elimination, and we accept without reserve the explication arrived at by the council of physicians, viz: "That this child must be ranged in the category of those of Hemorrhagic Diathesis. We will add further that this disposition to hemorrhage, seems to have originated in this child, and could not therefore, have been forseen, inasmuch as the parents showed no appearance of it, and the child was their first-born."

Be this as it may, this case seems to us to contain certain lessons, and we have believed it our duty to cite it here, in treating the question of the precocious eruption of the temporary teeth, and the afflictions with which it may be complicated. But before speaking of precocious eruptions, we think we ought to determine the average time of normal eruptions.

First dentition, it is generally believed, commences about the sixth month after birth. All observers have been unanimous in giving this as the average time for the appearance of the first tooth; it is the opinion of Trouseau. But it will be seen after numerous researches on this subject, that we place the average time at the seventh month. Of five hundred births, which we have observed, we have noticed the eruption of the inferior central incisors at the following periods:

| | | | | | |
|---|---|---|---|---|---|
| At birth | 1 | case. | At 7th month | 105 | cases. |
| At 1st month | 2 | " | At 8th " | 88 | " |
| At 2nd " | 3 | " | At 9th " | 49 | " |
| At 3rd " | 9 | " | At 10th " | 89 | " |
| At 4th " | 10 | " | At 11th " | 38 | " |
| At 5th " | 39 | " | At 12th " | 12 | " |
| At 6th " | 45 | " | At 2nd year | 10 | " |
| | | | Total | 500. | |

Doctor Bensenger, of Moscow, in a statement founded on the examination of a certain number of subjects of another race than ours, arrives at results nearly analogous to our own. But the most remarkable and interesting fact consists in the eruption of one or several teeth at birth.

Among the ancients there was a wide-spread superstition, that great destinies awaited male children born with erupted teeth, whilst with the females it was regarded as a sign of ill-omen. Pliny reports two cases of this kind. Louis XIV, and Mirabeau are said to have been born with teeth. In our day facts of this nature are well authenticated. In a record of births made at the Maternite of Paris, during a period of ten years (from 1858 to 1868) among 17,578 infants, three were born with teeth. Other cases have been observed by M. M. Sappey and Thore, Giraldis, Masse, Tannier and Gueniot, and by our friend M. Andre Sanson, in his own son born with two inferior central incisors. We, ourselves, have met with two cases; the first in 1860, in Pas de Calais, in a male infant, well developed—born at a regular period, with two inferior central incisors. The second is the one of which we have spoken above, and yet in the latter case, the eruption was not effected until the second day.

Authentic cases then exist of the eruption of teeth at birth. M. Blot, not having met with any in more that 20,000 births, is still doubtful on this point. On the other hand M. M. Besnier and Gueniot, believe them quite familiar to accoucheurs. These two opinions appear to us equally exaggerated; nevertheless, we must agree with M. Blot, that these cases are very rarely observed carefully, and that the people, and even physicians are often deceived in supposing teeth to be present, where it is only the natural hardness of the gum at the time of birth. In conceding that these cases are after all of a very limited number, it must nevertheless be admitted that science is possessed of facts enough to establish their existence beyond a doubt. M. Mattie, who had for a long time shared the opinion of M. Blot, has just observed a case which consequently re-establishes the fact. It is certain, however, that in all the examples collected, none have included the entire set, but of one or two only; oftener these are the lower central incisors.

---

### Part Second Extraction of Precocious, Temporary Teeth.

The causes of precocious eruption, escape us entirely. We cannot agree with certain authors, that it is due to the super-vital functional activity possessed by robust constitutions, for the reason that this influence does not manifest itself throughout the whole dental system. On the other hand, there are cases of precocity, observed among debilitated children, or those with syphilitic and scrofulous diathesis, etc.

Has not Trousseau affirmed, that eruption is more precocious with girls than with boys? Ordinarily, the teeth coming before their time, physiologically complete their development as regular as the others. They take little by little all the characteristics of the series to which they belong, * so that when the first dentition is completed, there is nothing to distinguish them from the

---

* These teeth do not, of course, "take little by little all the characteristics of the series to which they belong." This would be assuming that they did not possess all these when first developed and erupted.—Tr.

others, their color, form and position are normal, and their subsequent shedding occurs at the usual time, without influencing the eruption of the permanent teeth. If then they are extracted the dental apparatus of the child is incomplete during its early life, and a vacant space in the alveolar arch denotes their absence. Often, however, the neighboring teeth approach each other, nearly filling up this space, but even then it is easy to determine their absence, by counting the number remaining in the dental arch.

These considerations lead us to conclude that teeth which are erupted at birth, or before the seventh month, belong to the first dentition, and have nothing peculiar except their precocious eruption. * We must not then, by a wrong interpretation of this phenomenon, class these among the supernumerary teeth on account of their premature eruption.

Man has only two sets of teeth; this is a law to which we have not yet been able to find a single exception. All the cases of supplimentary dentition reported by various authors, are only cases of early or late eruptions. †

We believe we have dwelt sufficiently upon the fact of precocious eruption, and upon the interpretation that may be given to it. Nevertheless this anomaly of the dental system merits our attention, on account of the accidents with which it may be involved. We would not speak here of the ordinary maladies attending dentition, (local phenomena, inflammation, reflex and sympathetic disturbances, etc,) which may occur also as well in late as normal eruptions. What we would bring to your notice, and which is immediately connected with the foregoing observations, is a class of maladies that we will call on account of their situation *intra-follicular.* Like precocious eruption, these accidental maladies find their explication in some nutritive trouble,

---

* Had M. David been a dentist, he would not have considered this caution necessary, especially if he had written for dentists; for there has been no question among the members of our profession, that I am aware of, as to the classification of these teeth—all agreeing that they belong to the first dentition.—Tr.

† Though rarely met with, quite a number of cases of third dentition have been reported by reliable observers. And though entire sets are reported in the German Ephemerides, yet these third eruptions are usually of scattering teeth, imperfect in their organization of short duration, and almost or entirely useless.

which effects the elements in the formation of the dental organs. They manifest themselves in the dental follicules by disorders more or less grave, and sometimes causing their destruction. In some cases the follicular sac may be inflamed, and become the seat of a bloody or purulent discharge, become gangrenous, ulcerate, and give issue to its contents.

It was thus in a case observed by M. Mosse, of the precocious eruption of two inferior central incisors, in which the two crowns were eliminated through the ulceration of the follicular walls. In a case reported by M. Gueniot, the inflammation resulted in a true abscess, which was followed by a rupture of the wall, and the falling out of the crown. In a new born syphilitic child, that died eight days after birth, M. Perier, discovered in the interior of a follicule, protruding beneath the mucus membrane a bloody effusion, (hematocele of the dental follicule.) In simpler cases, and in the absence of all inflammatory symptoms it sometimes happens, that one or several follicules are thrown out, and found floating in the buccal cavity, still attached to the maxilla by a kind of pedicle.

For the guidance of practitioners, who may meet with cases of eruption at birth, we do not hesitate to declare that the extraction of teeth under these circumstances, as advised by certain authors, and adopted they say, by English practitioners, in our opinion would be altogether objectionable. Besides no serious argument can be advanced in its favor, and except in cases like the one reported in the beginning of this treatise, the trouble or inconvenience that such an anomaly can cause the child or the nurse, seems to us merely trivial. They are certainly very insignificant compared with the risk of an operation, of which the least consequence is to expose the new born infant to all the dangers of extraction, and to deprive it during the earlier period of childhood of organs, which are essential to the regular functions of the dental apparatus.

